# Tetra­methyl 1,4-dimethyl-13,14-dioxa­penta­cyclo­[8.2.1.1^4,7^.0^2,9^.0^3,8^]tetra­deca-5,11-diene-5,6,11,12-tetra­carboxyl­ate

**DOI:** 10.1107/S1600536812039219

**Published:** 2012-09-22

**Authors:** Alan J. Lough, Kelsey Jack, William Tam

**Affiliations:** a Department of Chemistry, University of Toronto, Toronto, Ontario, Canada M5S 3H6; b Department of Chemistry, University of Guelph, Guelph, Ontario, Canada N1G 2W1

## Abstract

In the title compound, C_22_H_24_O_14_, the relative stereochemistry at the cyclo­butane ring is *cis-anti-cis* and the methyl groups in the bicyclic rings are *syn* to each other. The two carboxyl­ate groups attached to the same —C=C— bond are disordered over two sets of sites in a 0.603 (2):0.397 (2) ratio. In the crystal, weak C—H⋯O hydrogen bonds connect mol­ecules into *C*(12) chains along [001] incorporating *R*_2_^2^_2_(10) rings.

## Related literature

For related structures, see: Lough *et al.* (2012*a* ▶,*b* ▶). For the synthetic background, see: Ballantine *et al.* (2009 ▶). For hydrogen-bond motifs, see: Bernstein *et al.* (1995 ▶).
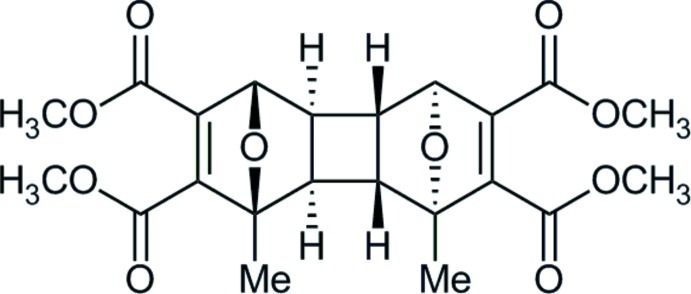


## Experimental

### 

#### Crystal data

C_22_H_24_O_10_
*M* *_r_* = 448.41
Orthorhombic, 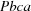

*a* = 11.5170 (14) Å
*b* = 13.9586 (15) Å
*c* = 26.413 (3) Å
*V* = 4246.1 (8) Å^3^
*Z* = 8
Mo *K*α radiation
μ = 0.11 mm^−1^
*T* = 147 K
0.33 × 0.22 × 0.06 mm

#### Data collection

Bruker Kappa APEX DUO CCD diffractometer
Absorption correction: multi-scan (Bruker, 2007 ▶) *T*_min_ = 0.964, *T*_max_ = 0.993
19411 measured reflections
4850 independent reflections
2974 reflections with *I* > 2σ(*I*)
*R*_int_ = 0.055

#### Refinement

*R*[*F* ^2^ > 2σ(*F* ^2^)] = 0.042
*wR*(*F* ^2^) = 0.096
*S* = 0.90
4850 reflections
322 parameters
32 restraints
H-atom parameters constrained
Δρ_max_ = 0.26 e Å^−3^
Δρ_min_ = −0.20 e Å^−3^

### 

Data collection: *APEX2* (Bruker, 2007 ▶); cell refinement: *SAINT* (Bruker, 2007 ▶); data reduction: *SAINT*; program(s) used to solve structure: *SHELXS97* (Sheldrick, 2008 ▶); program(s) used to refine structure: *SHELXL97* (Sheldrick, 2008 ▶); molecular graphics: *PLATON* (Spek, 2009 ▶); software used to prepare material for publication: *SHELXTL* (Sheldrick, 2008 ▶).

## Supplementary Material

Crystal structure: contains datablock(s) global, I. DOI: 10.1107/S1600536812039219/hb6952sup1.cif

Structure factors: contains datablock(s) I. DOI: 10.1107/S1600536812039219/hb6952Isup2.hkl

Supplementary material file. DOI: 10.1107/S1600536812039219/hb6952Isup3.cml

Additional supplementary materials:  crystallographic information; 3D view; checkCIF report

## Figures and Tables

**Table 1:** Hydrogen-bond geometry (Å, °)

*D*—H⋯*A*	*D*—H	H⋯*A*	*D*⋯*A*	*D*—H⋯*A*
C10—H10*A*⋯O2^i^	1.00	2.45	3.332 (2)	146
C14—H14*C*⋯O7^ii^	0.98	2.48	3.063 (3)	118

Symmetry codes: (i) 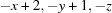; (ii) 
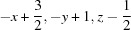
.

**Fig. 1.**
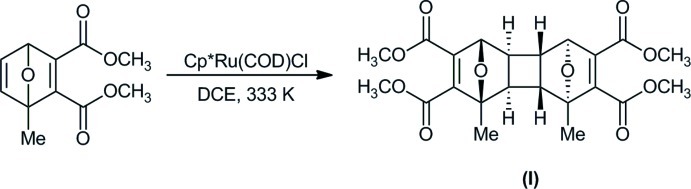
Reaction scheme

**Fig. 2.**
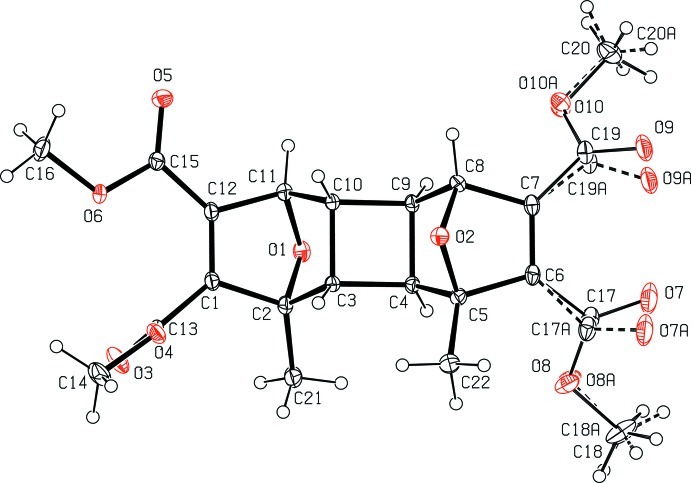
The molecular structure of (I) showing 30% probability ellipsoids. The bonds of the minor component of disorder are shown with dashed lines.

**Fig. 3.**
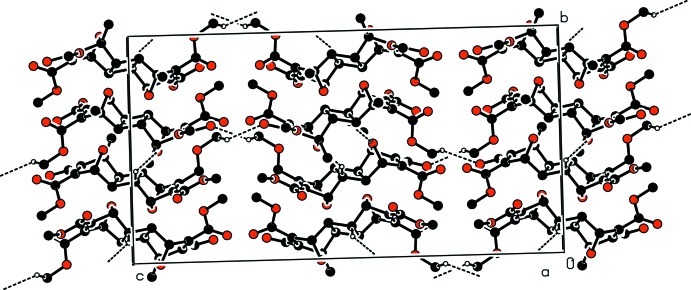
Part of the crystal structure showing weak hydrogen bonds as dashed lines. The disorder is not shown.

## supplementary crystallographic information

### Comment

We have recently investigated the Ru-catalyzed isomerization and dimerization reaction of oxanorbornadiene compounds (Ballantine *et al.*, 2009). When dissolved in 1,2-dichloroethane in the presence of Cp*Ru(COD)Cl, 1-methyl-2,3-dimethyl-7-oxabicyclo[2,2,1]hepta-2,5-diene-2,3-dicarboxylate will dimerize into (I) (Fig. 1). The desired product was resolved using fractional crystallization in methanol. The stereochemistry and regioschemistry of the product was determined by this single-crystal X-ray analysis. The only dimer product obtained was found to have a *cis*-anti-*cis* stereochemistry at the cyclobutane ring of the dimer, and the two Me groups in the bicyclic rings were found to be *syn* to each other.

The molecular structure of (I) is shown in Fig. 2. Two of the carboxylate groups attached to the same –C═C– bond were refined as disordered over two sets of sites with refined occupancies 0.603 (2) and 0.397 (2). In the crystal, weak C—H···O hydrogen bonds connect molecules into C(12) chains (Bernstein *et al.*, 1995) along [001] (Fig. 3) incorporating *R*^2^_2_(10) rings.

Related structures (Lough *et al.*, 2012*a*,*b*) are reported in the two following papers.

### Experimental

1-Methyl-2,3-dimethyl-7-oxabicyclo[2,2,1]hepta-2,5-diene-2,3-dicarboxylate (45 mg, 0.20 mmol) was weighed into an oven-dried vial, purged with nitrogen and transferred into a Dry Box. In the Dry Box, Cp*Ru(COD)Cl (10 mol%) was added to another oven dried vial and dissolved in 1,2-dichloroethane (0.3 ml). The Ru-catalyst was then transferred into the vial containing the 7-oxanorbornadiene. The vial was sealed with a screw cap and removed from the Dry Box. The reaction was heated at 333 K with stirring for 18 h. The crude product was purified by column chromatography (EtOAc:hexanes=2:3) followed by recrystallization in hexanes to give the dimer (I). Slow evapotation of a solution of (I) in hexanes gave colourless plates.

### Refinement

Hydrogen atoms were placed in calculated positions with C—H distances of 0.98 and 1.00 Å. They were included in the refinement in a riding-model approximation with *U*_iso_(H) = 1.2*U*_eq_(C) or 1.5*U*_eq_(C_methyl_). Constraints were applied to both the geometry and displacement parameters of the atoms in the disorder components using the FLAT, SADI and EADP instructions in *SHELXL* (Sheldrick, 2008).

### Crystal data

**Table:**

C_22_H_24_O_10_	*F*(000) = 1888
*M**_r_* = 448.41	*D*_x_ = 1.403 Mg m^−^^3^
Orthorhombic, *P**b**c**a*	Mo *K*α radiation, λ = 0.71073 Å
Hall symbol: -P 2ac 2ab	Cell parameters from 5611 reflections
*a* = 11.5170 (14) Å	θ = 2.4–27.4°
*b* = 13.9586 (15) Å	µ = 0.11 mm^−^^1^
*c* = 26.413 (3) Å	*T* = 147 K
*V* = 4246.1 (8) Å^3^	Plate, colourless
*Z* = 8	0.33 × 0.22 × 0.06 mm

### Data collection

**Table:**

Bruker Kappa APEX DUO CCD diffractometer	4850 independent reflections
Radiation source: fine-focus sealed tube	2974 reflections with *I* > 2σ(*I*)
Bruker Triumph monochromator	*R*_int_ = 0.055
φ and ω scans	θ_max_ = 27.5°, θ_min_ = 1.5°
Absorption correction: multi-scan (*SADABS*; Bruker, 2007)	*h* = −14→14
*T*_min_ = 0.964, *T*_max_ = 0.993	*k* = −10→18
19411 measured reflections	*l* = −34→18

### Refinement

**Table:**

Refinement on *F*^2^	Primary atom site location: structure-invariant direct methods
Least-squares matrix: full	Secondary atom site location: difference Fourier map
*R*[*F*^2^ > 2σ(*F*^2^)] = 0.042	Hydrogen site location: inferred from neighbouring sites
*wR*(*F*^2^) = 0.096	H-atom parameters constrained
*S* = 0.90	*w* = 1/[σ^2^(*F*_o_^2^) + (0.0451*P*)^2^] where *P* = (*F*_o_^2^ + 2*F*_c_^2^)/3
4850 reflections	(Δ/σ)_max_ = 0.001
322 parameters	Δρ_max_ = 0.26 e Å^−^^3^
32 restraints	Δρ_min_ = −0.20 e Å^−^^3^

### Special details

**Table:**

Geometry. All e.s.d.'s (except the e.s.d. in the dihedral angle between two l.s. planes) are estimated using the full covariance matrix. The cell e.s.d.'s are taken into account individually in the estimation of e.s.d.'s in distances, angles and torsion angles; correlations between e.s.d.'s in cell parameters are only used when they are defined by crystal symmetry. An approximate (isotropic) treatment of cell e.s.d.'s is used for estimating e.s.d.'s involving l.s. planes.
Refinement. Refinement of *F*^2^ against ALL reflections. The weighted *R*-factor *wR* and goodness of fit *S* are based on *F*^2^, conventional *R*-factors *R* are based on *F*, with *F* set to zero for negative *F*^2^. The threshold expression of *F*^2^ > σ(*F*^2^) is used only for calculating *R*-factors(gt) *etc*. and is not relevant to the choice of reflections for refinement. *R*-factors based on *F*^2^ are statistically about twice as large as those based on *F*, and *R*- factors based on ALL data will be even larger.

### Fractional atomic coordinates and isotropic or equivalent isotropic displacement parameters (Å^2^)

**Table:**

	*x*	*y*	*z*	*U*_iso_*/*U*_eq_	Occ. (<1)
O1	0.81775 (11)	0.24086 (8)	−0.04639 (4)	0.0228 (3)	
O2	0.87040 (10)	0.50450 (8)	0.06438 (4)	0.0202 (3)	
O3	0.77858 (13)	0.36303 (10)	−0.19294 (4)	0.0398 (4)	
O4	0.85192 (11)	0.49305 (9)	−0.15417 (4)	0.0263 (3)	
O5	1.15910 (11)	0.28111 (9)	−0.10455 (4)	0.0268 (3)	
O6	1.06300 (11)	0.35923 (9)	−0.16646 (4)	0.0269 (3)	
O7	0.6189 (2)	0.3994 (2)	0.18885 (7)	0.0503 (7)	0.603 (2)
O8	0.5320 (9)	0.4330 (6)	0.1139 (5)	0.0327 (14)	0.603 (2)
O9	0.8653 (3)	0.3827 (2)	0.22013 (8)	0.0435 (8)	0.603 (2)
O10	0.9341 (6)	0.2732 (5)	0.1670 (5)	0.0290 (14)	0.603 (2)
C17	0.6234 (3)	0.4210 (2)	0.14455 (12)	0.0264 (8)	0.603 (2)
C19	0.8778 (3)	0.3546 (3)	0.17727 (10)	0.0249 (6)	0.603 (2)
C18	0.4202 (12)	0.4196 (9)	0.1391 (6)	0.0454 (6)	0.603 (2)
H18A	0.3578	0.4217	0.1139	0.068*	0.603 (2)
H18B	0.4085	0.4706	0.1640	0.068*	0.603 (2)
H18C	0.4193	0.3573	0.1562	0.068*	0.603 (2)
C20	0.9776 (10)	0.2224 (9)	0.2114 (6)	0.0436 (9)	0.603 (2)
H20A	1.0204	0.1653	0.2006	0.065*	0.603 (2)
H20B	0.9123	0.2033	0.2329	0.065*	0.603 (2)
H20C	1.0295	0.2646	0.2306	0.065*	0.603 (2)
O7A	0.6002 (3)	0.4831 (3)	0.18225 (12)	0.0503 (7)	0.397 (2)
O8A	0.5296 (14)	0.4105 (11)	0.1152 (8)	0.0327 (14)	0.397 (2)
O9A	0.7972 (4)	0.3518 (3)	0.21445 (14)	0.0435 (8)	0.397 (2)
O10A	0.9507 (10)	0.2917 (9)	0.1692 (8)	0.0290 (14)	0.397 (2)
C17A	0.6182 (4)	0.4469 (4)	0.14155 (16)	0.0264 (8)	0.397 (2)
C19A	0.8570 (5)	0.3462 (4)	0.17719 (13)	0.0249 (6)	0.397 (2)
C18A	0.4166 (19)	0.4198 (14)	0.1398 (10)	0.0454 (6)	0.397 (2)
H18D	0.3558	0.3959	0.1171	0.068*	0.397 (2)
H18E	0.4020	0.4873	0.1477	0.068*	0.397 (2)
H18F	0.4163	0.3823	0.1712	0.068*	0.397 (2)
C20A	0.9845 (17)	0.2309 (13)	0.2116 (8)	0.0436 (9)	0.397 (2)
H20D	1.0628	0.2059	0.2058	0.065*	0.397 (2)
H20E	0.9297	0.1775	0.2146	0.065*	0.397 (2)
H20F	0.9836	0.2686	0.2429	0.065*	0.397 (2)
C1	0.85556 (16)	0.34792 (12)	−0.11010 (5)	0.0196 (4)	
C2	0.77038 (16)	0.32925 (12)	−0.06633 (5)	0.0200 (4)	
C3	0.80888 (15)	0.40228 (12)	−0.02535 (5)	0.0170 (4)	
H3A	0.8102	0.4708	−0.0364	0.020*	
C4	0.75885 (15)	0.38416 (12)	0.02843 (5)	0.0172 (4)	
H4A	0.6914	0.3392	0.0302	0.021*	
C5	0.74926 (15)	0.47494 (12)	0.06159 (5)	0.0190 (4)	
C6	0.73289 (15)	0.43738 (12)	0.11604 (5)	0.0200 (4)	
C7	0.83590 (16)	0.40288 (12)	0.13030 (5)	0.0201 (4)	
C8	0.91584 (16)	0.41679 (12)	0.08511 (5)	0.0190 (4)	
H8A	1.0007	0.4176	0.0931	0.023*	
C9	0.87811 (15)	0.34188 (12)	0.04533 (5)	0.0175 (4)	
H9A	0.8773	0.2737	0.0569	0.021*	
C10	0.92895 (15)	0.36020 (12)	−0.00825 (5)	0.0173 (4)	
H10A	0.9962	0.4054	−0.0098	0.021*	
C11	0.93701 (15)	0.27200 (12)	−0.04294 (5)	0.0204 (4)	
H11A	0.9934	0.2218	−0.0318	0.024*	
C12	0.95736 (16)	0.31197 (12)	−0.09594 (5)	0.0198 (4)	
C13	0.82498 (16)	0.40042 (13)	−0.15740 (5)	0.0221 (4)	
C14	0.83509 (19)	0.54896 (15)	−0.20023 (6)	0.0398 (5)	
H14A	0.8350	0.6174	−0.1919	0.060*	
H14B	0.7607	0.5317	−0.2157	0.060*	
H14C	0.8983	0.5354	−0.2240	0.060*	
C15	1.07098 (16)	0.31541 (12)	−0.12149 (5)	0.0194 (4)	
C16	1.16955 (17)	0.36413 (15)	−0.19566 (6)	0.0331 (5)	
H16A	1.1593	0.4087	−0.2240	0.050*	
H16B	1.1885	0.3004	−0.2088	0.050*	
H16C	1.2329	0.3867	−0.1739	0.050*	
C21	0.64336 (16)	0.31957 (14)	−0.07817 (6)	0.0292 (5)	
H21A	0.6325	0.2705	−0.1043	0.044*	
H21B	0.6134	0.3810	−0.0905	0.044*	
H21C	0.6013	0.3009	−0.0475	0.044*	
C22	0.67345 (17)	0.55607 (13)	0.04345 (6)	0.0271 (4)	
H22A	0.6715	0.6066	0.0692	0.041*	
H22B	0.5946	0.5323	0.0375	0.041*	
H22C	0.7051	0.5821	0.0119	0.041*	

### Atomic displacement parameters (Å^2^)

**Table:**

	*U*^11^	*U*^22^	*U*^33^	*U*^12^	*U*^13^	*U*^23^
O1	0.0276 (8)	0.0218 (7)	0.0189 (5)	−0.0031 (6)	0.0024 (5)	0.0019 (5)
O2	0.0201 (7)	0.0184 (6)	0.0221 (5)	−0.0020 (5)	0.0006 (5)	0.0023 (5)
O3	0.0495 (10)	0.0499 (9)	0.0200 (6)	−0.0059 (8)	−0.0140 (6)	−0.0024 (6)
O4	0.0292 (8)	0.0311 (8)	0.0185 (5)	−0.0006 (6)	−0.0042 (5)	0.0084 (5)
O5	0.0239 (8)	0.0290 (7)	0.0275 (6)	0.0066 (6)	−0.0018 (5)	−0.0016 (5)
O6	0.0217 (7)	0.0445 (8)	0.0145 (5)	0.0040 (6)	0.0054 (5)	0.0052 (5)
O7	0.0397 (13)	0.0895 (19)	0.0218 (9)	−0.0210 (16)	0.0109 (8)	−0.0115 (13)
O8	0.0193 (9)	0.037 (4)	0.0422 (9)	0.005 (2)	0.0050 (8)	0.001 (3)
O9	0.059 (2)	0.0560 (19)	0.0152 (8)	0.0237 (16)	0.0032 (13)	0.0002 (10)
O10	0.027 (2)	0.037 (3)	0.0225 (12)	0.007 (2)	−0.0007 (18)	0.009 (2)
C17	0.0282 (13)	0.029 (2)	0.0216 (10)	−0.0092 (13)	0.0044 (9)	−0.0074 (11)
C19	0.0325 (18)	0.0246 (12)	0.0175 (8)	−0.0020 (12)	0.0043 (9)	0.0009 (8)
C18	0.0207 (14)	0.0459 (14)	0.0696 (15)	0.0047 (11)	0.0146 (12)	−0.0102 (12)
C20	0.0403 (17)	0.055 (2)	0.0354 (10)	0.0094 (18)	−0.0040 (12)	0.0208 (13)
O7A	0.0397 (13)	0.0895 (19)	0.0218 (9)	−0.0210 (16)	0.0109 (8)	−0.0115 (13)
O8A	0.0193 (9)	0.037 (4)	0.0422 (9)	0.005 (2)	0.0050 (8)	0.001 (3)
O9A	0.059 (2)	0.0560 (19)	0.0152 (8)	0.0237 (16)	0.0032 (13)	0.0002 (10)
O10A	0.027 (2)	0.037 (3)	0.0225 (12)	0.007 (2)	−0.0007 (18)	0.009 (2)
C17A	0.0282 (13)	0.029 (2)	0.0216 (10)	−0.0092 (13)	0.0044 (9)	−0.0074 (11)
C19A	0.0325 (18)	0.0246 (12)	0.0175 (8)	−0.0020 (12)	0.0043 (9)	0.0009 (8)
C18A	0.0207 (14)	0.0459 (14)	0.0696 (15)	0.0047 (11)	0.0146 (12)	−0.0102 (12)
C20A	0.0403 (17)	0.055 (2)	0.0354 (10)	0.0094 (18)	−0.0040 (12)	0.0208 (13)
C1	0.0212 (10)	0.0244 (10)	0.0131 (7)	−0.0023 (8)	0.0007 (7)	−0.0009 (7)
C2	0.0200 (10)	0.0245 (10)	0.0154 (7)	−0.0012 (8)	0.0022 (7)	0.0018 (7)
C3	0.0158 (9)	0.0204 (9)	0.0149 (7)	−0.0001 (7)	0.0007 (6)	0.0033 (7)
C4	0.0166 (9)	0.0206 (9)	0.0144 (7)	−0.0027 (8)	0.0015 (6)	0.0000 (6)
C5	0.0169 (9)	0.0218 (9)	0.0182 (7)	−0.0023 (8)	0.0009 (7)	0.0004 (7)
C6	0.0243 (10)	0.0210 (9)	0.0148 (7)	−0.0039 (8)	0.0026 (7)	−0.0051 (7)
C7	0.0263 (11)	0.0189 (9)	0.0149 (7)	−0.0030 (8)	0.0012 (7)	−0.0037 (7)
C8	0.0197 (10)	0.0216 (10)	0.0158 (7)	0.0011 (8)	−0.0012 (7)	0.0030 (7)
C9	0.0207 (10)	0.0189 (9)	0.0128 (7)	−0.0001 (7)	0.0017 (6)	0.0021 (6)
C10	0.0170 (9)	0.0205 (9)	0.0144 (7)	0.0004 (8)	0.0014 (6)	0.0033 (7)
C11	0.0201 (10)	0.0244 (10)	0.0167 (7)	0.0027 (8)	0.0017 (7)	0.0025 (7)
C12	0.0235 (10)	0.0230 (10)	0.0127 (7)	0.0005 (8)	0.0016 (7)	−0.0008 (7)
C13	0.0187 (10)	0.0326 (11)	0.0149 (7)	0.0027 (8)	0.0032 (7)	0.0003 (7)
C14	0.0407 (14)	0.0500 (14)	0.0286 (9)	0.0003 (11)	−0.0041 (9)	0.0220 (9)
C15	0.0226 (11)	0.0201 (9)	0.0156 (7)	0.0021 (8)	−0.0001 (7)	−0.0042 (7)
C16	0.0278 (12)	0.0469 (13)	0.0245 (8)	−0.0030 (10)	0.0118 (8)	−0.0023 (8)
C21	0.0227 (11)	0.0459 (13)	0.0191 (8)	−0.0075 (10)	0.0002 (7)	−0.0012 (8)
C22	0.0269 (11)	0.0277 (11)	0.0266 (8)	0.0062 (9)	0.0004 (8)	0.0005 (8)

### Geometric parameters (Å, º)

**Table:**

O1—C11	1.443 (2)	C20A—H20F	0.9800
O1—C2	1.448 (2)	C1—C12	1.329 (2)
O2—C8	1.4396 (19)	C1—C13	1.490 (2)
O2—C5	1.457 (2)	C1—C2	1.539 (2)
O3—C13	1.200 (2)	C2—C21	1.502 (3)
O4—C13	1.332 (2)	C2—C3	1.552 (2)
O4—C14	1.4584 (19)	C3—C4	1.554 (2)
O5—C15	1.208 (2)	C3—C10	1.569 (2)
O6—C15	1.3393 (19)	C3—H3A	1.0000
O6—C16	1.451 (2)	C4—C5	1.544 (2)
O7—C17	1.210 (3)	C4—C9	1.560 (2)
O8—C17	1.338 (3)	C4—H4A	1.0000
O8—C18	1.461 (3)	C5—C22	1.508 (2)
O9—C19	1.207 (3)	C5—C6	1.542 (2)
O10—C19	1.337 (3)	C6—C7	1.335 (2)
O10—C20	1.458 (3)	C7—C8	1.520 (2)
C17—C6	1.486 (3)	C8—C9	1.545 (2)
C19—C7	1.492 (3)	C8—H8A	1.0000
C18—H18A	0.9800	C9—C10	1.553 (2)
C18—H18B	0.9800	C9—H9A	1.0000
C18—H18C	0.9800	C10—C11	1.537 (2)
C20—H20A	0.9800	C10—H10A	1.0000
C20—H20B	0.9800	C11—C12	1.525 (2)
C20—H20C	0.9800	C11—H11A	1.0000
O7A—C17A	1.206 (4)	C12—C15	1.473 (2)
O8A—C17A	1.337 (4)	C14—H14A	0.9800
O8A—C18A	1.460 (4)	C14—H14B	0.9800
O9A—C19A	1.203 (4)	C14—H14C	0.9800
O10A—C19A	1.337 (4)	C16—H16A	0.9800
O10A—C20A	1.459 (4)	C16—H16B	0.9800
C17A—C6	1.488 (4)	C16—H16C	0.9800
C19A—C7	1.490 (4)	C21—H21A	0.9800
C18A—H18D	0.9800	C21—H21B	0.9800
C18A—H18E	0.9800	C21—H21C	0.9800
C18A—H18F	0.9800	C22—H22A	0.9800
C20A—H20D	0.9800	C22—H22B	0.9800
C20A—H20E	0.9800	C22—H22C	0.9800
			
C11—O1—C2	97.18 (12)	C17A—C6—C5	120.0 (2)
C8—O2—C5	97.27 (12)	C6—C7—C19A	124.8 (3)
C13—O4—C14	115.79 (13)	C6—C7—C19	133.23 (19)
C15—O6—C16	115.78 (14)	C6—C7—C8	105.71 (12)
C17—O8—C18	113.7 (10)	C19A—C7—C8	128.5 (3)
C19—O10—C20	114.7 (8)	C19—C7—C8	121.00 (19)
O7—C17—O8	125.6 (7)	O2—C8—C7	100.78 (13)
O7—C17—C6	124.4 (3)	O2—C8—C9	102.40 (12)
O8—C17—C6	110.0 (7)	C7—C8—C9	106.10 (13)
O9—C19—O10	121.6 (6)	O2—C8—H8A	115.3
O9—C19—C7	126.5 (3)	C7—C8—H8A	115.3
O10—C19—C7	111.9 (6)	C9—C8—H8A	115.3
C17A—O8A—C18A	114.4 (17)	C8—C9—C10	113.73 (13)
C19A—O10A—C20A	115.1 (14)	C8—C9—C4	100.73 (13)
O7A—C17A—O8A	119.5 (12)	C10—C9—C4	90.51 (11)
O7A—C17A—C6	126.5 (5)	C8—C9—H9A	116.0
O8A—C17A—C6	114.0 (11)	C10—C9—H9A	116.0
O9A—C19A—O10A	128.9 (10)	C4—C9—H9A	116.0
O9A—C19A—C7	123.5 (5)	C11—C10—C9	115.72 (13)
O10A—C19A—C7	107.6 (10)	C11—C10—C3	100.46 (13)
O8A—C18A—H18D	109.5	C9—C10—C3	89.52 (12)
O8A—C18A—H18E	109.5	C11—C10—H10A	115.7
H18D—C18A—H18E	109.5	C9—C10—H10A	115.7
O8A—C18A—H18F	109.5	C3—C10—H10A	115.7
H18D—C18A—H18F	109.5	O1—C11—C12	101.45 (12)
H18E—C18A—H18F	109.5	O1—C11—C10	102.78 (13)
O10A—C20A—H20D	109.5	C12—C11—C10	105.27 (13)
O10A—C20A—H20E	109.5	O1—C11—H11A	115.2
H20D—C20A—H20E	109.5	C12—C11—H11A	115.2
O10A—C20A—H20F	109.5	C10—C11—H11A	115.2
H20D—C20A—H20F	109.5	C1—C12—C15	129.97 (14)
H20E—C20A—H20F	109.5	C1—C12—C11	105.13 (14)
C12—C1—C13	129.05 (15)	C15—C12—C11	124.67 (15)
C12—C1—C2	106.68 (13)	O3—C13—O4	125.16 (15)
C13—C1—C2	124.23 (15)	O3—C13—C1	123.18 (17)
O1—C2—C21	111.47 (14)	O4—C13—C1	111.62 (14)
O1—C2—C1	100.22 (13)	O4—C14—H14A	109.5
C21—C2—C1	118.67 (13)	O4—C14—H14B	109.5
O1—C2—C3	101.44 (12)	H14A—C14—H14B	109.5
C21—C2—C3	118.86 (15)	O4—C14—H14C	109.5
C1—C2—C3	103.33 (13)	H14A—C14—H14C	109.5
C2—C3—C4	115.13 (14)	H14B—C14—H14C	109.5
C2—C3—C10	101.93 (13)	O5—C15—O6	124.55 (16)
C4—C3—C10	90.16 (11)	O5—C15—C12	124.31 (15)
C2—C3—H3A	115.4	O6—C15—C12	111.12 (15)
C4—C3—H3A	115.4	O6—C16—H16A	109.5
C10—C3—H3A	115.4	O6—C16—H16B	109.5
C5—C4—C3	114.29 (13)	H16A—C16—H16B	109.5
C5—C4—C9	102.18 (13)	O6—C16—H16C	109.5
C3—C4—C9	89.81 (12)	H16A—C16—H16C	109.5
C5—C4—H4A	115.7	H16B—C16—H16C	109.5
C3—C4—H4A	115.7	C2—C21—H21A	109.5
C9—C4—H4A	115.7	C2—C21—H21B	109.5
O2—C5—C22	110.97 (14)	H21A—C21—H21B	109.5
O2—C5—C6	99.57 (12)	C2—C21—H21C	109.5
C22—C5—C6	118.73 (14)	H21A—C21—H21C	109.5
O2—C5—C4	101.11 (13)	H21B—C21—H21C	109.5
C22—C5—C4	118.51 (13)	C5—C22—H22A	109.5
C6—C5—C4	104.99 (13)	C5—C22—H22B	109.5
C7—C6—C17	123.8 (2)	H22A—C22—H22B	109.5
C7—C6—C17A	133.9 (2)	C5—C22—H22C	109.5
C7—C6—C5	106.09 (14)	H22A—C22—H22C	109.5
C17—C6—C5	128.9 (2)	H22B—C22—H22C	109.5
			
C18—O8—C17—O7	1.3 (6)	C17A—C6—C7—C8	−179.3 (3)
C18—O8—C17—C6	−179.2 (4)	C5—C6—C7—C8	−0.99 (17)
C20—O10—C19—O9	0.2 (4)	O9A—C19A—C7—C6	−25.8 (5)
C20—O10—C19—C7	179.9 (2)	O10A—C19A—C7—C6	154.4 (5)
C18A—O8A—C17A—O7A	0.3 (6)	O9A—C19A—C7—C19	121 (2)
C18A—O8A—C17A—C6	−179.8 (4)	O10A—C19A—C7—C19	−59 (2)
C20A—O10A—C19A—O9A	0.1 (4)	O9A—C19A—C7—C8	167.3 (4)
C20A—O10A—C19A—C7	179.9 (2)	O10A—C19A—C7—C8	−12.5 (4)
C11—O1—C2—C21	176.12 (12)	O9—C19—C7—C6	−51.3 (4)
C11—O1—C2—C1	49.62 (12)	O10—C19—C7—C6	129.0 (4)
C11—O1—C2—C3	−56.37 (13)	O9—C19—C7—C19A	−89 (2)
C12—C1—C2—O1	−31.08 (16)	O10—C19—C7—C19A	91.3 (19)
C13—C1—C2—O1	151.18 (16)	O9—C19—C7—C8	132.0 (3)
C12—C1—C2—C21	−152.58 (16)	O10—C19—C7—C8	−47.6 (3)
C13—C1—C2—C21	29.7 (2)	C5—O2—C8—C7	−51.98 (13)
C12—C1—C2—C3	73.39 (17)	C5—O2—C8—C9	57.35 (13)
C13—C1—C2—C3	−104.35 (18)	C6—C7—C8—O2	33.57 (16)
O1—C2—C3—C4	−62.06 (17)	C19A—C7—C8—O2	−157.5 (3)
C21—C2—C3—C4	60.5 (2)	C19—C7—C8—O2	−148.9 (2)
C1—C2—C3—C4	−165.59 (14)	C6—C7—C8—C9	−72.84 (16)
O1—C2—C3—C10	33.81 (14)	C19A—C7—C8—C9	96.0 (3)
C21—C2—C3—C10	156.36 (14)	C19—C7—C8—C9	104.6 (2)
C1—C2—C3—C10	−69.72 (15)	O2—C8—C9—C10	60.81 (17)
C2—C3—C4—C5	−153.67 (15)	C7—C8—C9—C10	166.05 (13)
C10—C3—C4—C5	103.05 (15)	O2—C8—C9—C4	−34.53 (14)
C2—C3—C4—C9	103.03 (15)	C7—C8—C9—C4	70.71 (14)
C10—C3—C4—C9	−0.25 (12)	C5—C4—C9—C8	−0.31 (14)
C8—O2—C5—C22	176.56 (12)	C3—C4—C9—C8	114.53 (12)
C8—O2—C5—C6	50.64 (13)	C5—C4—C9—C10	−114.60 (12)
C8—O2—C5—C4	−56.84 (12)	C3—C4—C9—C10	0.25 (12)
C3—C4—C5—O2	−60.95 (16)	C8—C9—C10—C11	156.40 (14)
C9—C4—C5—O2	34.43 (13)	C4—C9—C10—C11	−101.64 (15)
C3—C4—C5—C22	60.5 (2)	C8—C9—C10—C3	−102.21 (15)
C9—C4—C5—C22	155.88 (15)	C4—C9—C10—C3	−0.25 (12)
C3—C4—C5—C6	−164.12 (14)	C2—C3—C10—C11	0.57 (14)
C9—C4—C5—C6	−68.74 (15)	C4—C3—C10—C11	116.34 (12)
O7—C17—C6—C7	21.3 (4)	C2—C3—C10—C9	−115.52 (12)
O8—C17—C6—C7	−158.1 (4)	C4—C3—C10—C9	0.25 (12)
O7—C17—C6—C17A	−116.9 (14)	C2—O1—C11—C12	−50.97 (13)
O8—C17—C6—C17A	63.7 (13)	C2—O1—C11—C10	57.78 (12)
O7—C17—C6—C5	−173.1 (2)	C9—C10—C11—O1	59.43 (16)
O8—C17—C6—C5	7.5 (3)	C3—C10—C11—O1	−35.13 (14)
O7A—C17A—C6—C7	51.7 (6)	C9—C10—C11—C12	165.26 (14)
O8A—C17A—C6—C7	−128.1 (6)	C3—C10—C11—C12	70.70 (15)
O7A—C17A—C6—C17	101.9 (14)	C13—C1—C12—C15	2.2 (3)
O8A—C17A—C6—C17	−77.9 (14)	C2—C1—C12—C15	−175.43 (17)
O7A—C17A—C6—C5	−126.4 (4)	C13—C1—C12—C11	176.74 (17)
O8A—C17A—C6—C5	53.8 (5)	C2—C1—C12—C11	−0.86 (18)
O2—C5—C6—C7	−31.31 (16)	O1—C11—C12—C1	32.81 (17)
C22—C5—C6—C7	−151.73 (16)	C10—C11—C12—C1	−74.00 (17)
C4—C5—C6—C7	73.01 (17)	O1—C11—C12—C15	−152.24 (15)
O2—C5—C6—C17	161.13 (19)	C10—C11—C12—C15	100.94 (18)
C22—C5—C6—C17	40.7 (3)	C14—O4—C13—O3	−7.5 (3)
C4—C5—C6—C17	−94.5 (2)	C14—O4—C13—C1	174.64 (15)
O2—C5—C6—C17A	147.2 (3)	C12—C1—C13—O3	99.2 (2)
C22—C5—C6—C17A	26.8 (3)	C2—C1—C13—O3	−83.6 (2)
C4—C5—C6—C17A	−108.4 (3)	C12—C1—C13—O4	−82.9 (2)
C17—C6—C7—C19A	−2.0 (3)	C2—C1—C13—O4	94.3 (2)
C17A—C6—C7—C19A	11.3 (5)	C16—O6—C15—O5	0.1 (2)
C5—C6—C7—C19A	−170.4 (3)	C16—O6—C15—C12	−178.24 (14)
C17—C6—C7—C19	−9.7 (4)	C1—C12—C15—O5	178.80 (18)
C17A—C6—C7—C19	3.7 (5)	C11—C12—C15—O5	5.2 (3)
C5—C6—C7—C19	−178.0 (3)	C1—C12—C15—O6	−2.9 (3)
C17—C6—C7—C8	167.37 (18)	C11—C12—C15—O6	−176.48 (15)

### Hydrogen-bond geometry (Å, º)

**Table:**

*D*—H···*A*	*D*—H	H···*A*	*D*···*A*	*D*—H···*A*
C10—H10*A*···O2^i^	1.00	2.45	3.332 (2)	146
C14—H14*C*···O7^ii^	0.98	2.48	3.063 (3)	118

Symmetry codes: (i) −*x*+2, −*y*+1, −*z*; (ii) −*x*+3/2, −*y*+1, *z*−1/2.
